# Immunoglobulin GM Genes, Cytomegalovirus Immunoevasion, and the Risk of Glioma, Neuroblastoma, and Breast Cancer

**DOI:** 10.3389/fonc.2014.00236

**Published:** 2014-08-29

**Authors:** Janardan P. Pandey

**Affiliations:** ^1^Department of Microbiology and Immunology, Medical University of South Carolina, Charleston, SC, USA

**Keywords:** GM allotypes, immunoevasion, cytomegalovirus, glioma, neuroblastoma, breast cancer

## Abstract

Human cytomegalovirus (HCMV), a common herpes virus, has been reported to be a risk factor for many diseases, including malignant diseases such as glioma, neuroblastoma, and breast cancer. Some of the HCMV-associated diseases (e.g., glioma) are rare. The question arises: how could a common virus be associated with uncommon diseases? Interactions between a major gene complex of the human immune system and a viral immunoevasion strategy – a probable mechanism of their co-evolutionary adaptation – may shed light on this paradox. To ensure its survival, HCMV has evolved sophisticated immunoevasion strategies. One strategy involves encoding decoy Fcγ receptors (FcγR), which may enable the virus to evade host immunosurveillance by avoiding the Fcγ-mediated effector consequences of anti-HCMV IgG antibody binding. Immunoglobulin G1 proteins expressing GM (γ marker) alleles 3 and 17 have differential affinity to the HCMV *TRL11/IRL11*-encoded FcγR, and thus act as effect modifiers of HCMV-associated malignancies. The high affinity GM 3 allele has been shown to be a risk factor for neuroblastoma, glioma, and breast cancer. Additional studies involving other viral FcγRs as well as GM alleles expressed on other IgG subclasses are warranted.

## Introduction

**Figure 1:**
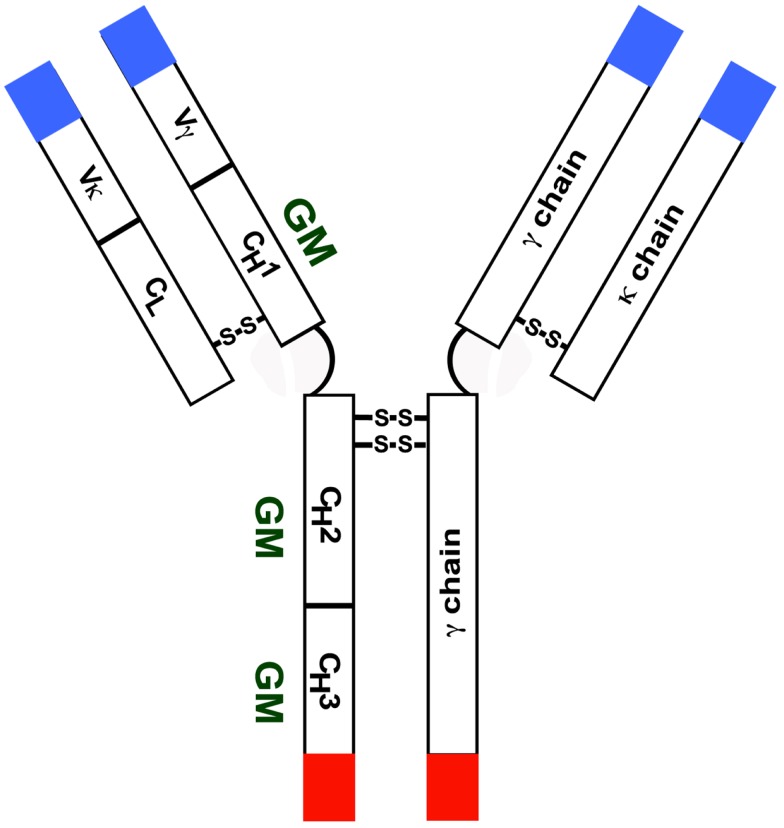
**Diagrammatic representation of an IgG molecule with a κ light chain**. Most GM alleles are expressed in the CH2 and CH3 domains.

Immunoglobulin (Ig) GM (γ marker) allotypes are encoded by three very closely linked genes – Ig heavy chain G1 (*IGHG1*), *IGHG2*, and *IGHG3* – on chromosome 14q32. There are currently 18 serologically testable GM specificities – four on γ1 (1/a, 2/x, 3/f, 17/z), one on γ2 (23/n), and 13 on γ3 (5/b1, 6/c3, 10/b5, 11/b0, 13/b3, 14/b4, 15/s, 16/t, 21/g1, 24/c5, 26/u, 27/v, 28/g5). Ig γ4 chains do not express γ4-specific unique allotypes, but they do express isoallotypes – determinants that behave as alleles in one IgG subclass (allotypes) but are also expressed in all molecules of at least one other subclass (isotypes). With the exception of allelic GM 3 and GM 17 determinants expressed in the Fd region, all other GM alleles are expressed in the Fc region of γ chains (Figure 1). Linkage disequilibrium in the GM gene complex within a racial group is almost absolute and the determinants are transmitted as a group – haplotypes. Each major race has a distinct array of several GM haplotypes (1–3). There are also qualitative differences in the distribution of GM allotypes among various racial/ethnic groups. For instance, GM 3 is not commonly found in people of African descent or GM 6 in those of European ancestry; GM 1 is polymorphic only in people of European ancestry.

Population genetic properties of the GM gene complex – marked differences in allele frequencies of among races, strong linkage disequilibrium within a race, and racially restricted occurrence of GM haplotypes – collectively suggest that differential selection over many generations may have played an important role in the maintenance of polymorphism at GM loci. As first suggested by Haldane (4) and recently emphasized by others (5), major infectious diseases have been the principal selective forces of natural selection in humans. GM allotypes, as likely targets of these selective forces, could contribute to the outcome of infection via allotype-restricted differential immunity to the infectious pathogens.

This mini review will discuss how GM alleles influence certain immunoevasion strategies of human cytomegalovirus (HCMV) and thus act as potential effect modifiers of some HCMV-associated malignant diseases. Other putative mechanisms of GM gene involvement in immunity to self and non-self antigens are also discussed.

## GM Alleles and Cytomegalovirus Immunoevasion

Human cytomegalovirus has evolved highly sophisticated strategies for evading host immunosurveillance. One strategy involves generating proteins that have functional properties of the Fcγ receptor (FcγR) (6), which may enable the virus to evade host immunosurveillance by avoiding the effector consequences of anti-HCMV IgG antibody binding, such as antibody-dependent cellular cytotoxicity (ADCC), antibody-dependent cellular phagocytosis, and antibody-dependent complement-dependent cytotoxicity. The HCMV-encoded FcγR may interfere with Fcγ-mediated effector functions by bipolar bridging, in which the Fab part of the anti-HCMV IgG antibody (paratope) binds to its antigenic target (epitope) on the virus, whereas the Fcγ part of the antibody binds to the FcγR-like binding site on the viral protein, thus offering survival advantage to the virus by sterically hindering the access of (cellular) FcγR-expressing effector cells to the HCMV-infected cells.

Certain GM alleles appear to modulate this immunoevasion strategy of HCMV (7). We have shown that HCMV *TRL11/IRL11*-encoded FcγR has significantly higher affinity for IgG1 proteins expressing the GM 3+,1−,2−allotypes than for those expressing the allelic GM 17+,1+,2+ allotypes (*p* = 0.0005). Higher affinity of GM 3+,1−,2−expressing IgG1 to the *TRL11*-encoded FcγR would imply that subjects with GM 3+,1−,2−allotypes would be more likely to have their Fc domains scavenged, thereby reducing their immunological competence to eliminate the virus through ADCC and other Fc-mediated effector mechanisms. Consequently, subjects possessing the GM 3 + ,1−,2− allotypes would be expected to be at an increased risk – while those carrying the GM 17+,1+,2+ allotypes (because of the lower affinity to the viral FcγR) at a reduced risk – of developing HCMV-associated diseases. These results are reminiscent of those reported for the herpes simplex virus type 1 (HSV1)-encoded FcγR; the binding pattern, however, is reversed: the HSV1-encoded FcγR binds much more strongly to the IgG molecule carrying the GM 1,17 allele than the one carrying the GM 3 allele (8). The contrasting binding patterns of the two viral FcγRs shed light on the nature of the evolutionary mechanism that maintains genetic polymorphism at the γ1 locus. Since IgG antibodies expressing both alleles/haplotypes would be expected to be protective factors due to their modulating effects against the immunoevasion strategies of HCMV and HSV1, the heterozygotes at this locus would have advantage over homozygotes, resulting in the persistence of both alleles/haplotypes in the population. This would represent an example of balancing selection for the human adaptation of herpes viruses.

A brief review of the studies that suggest a role for GM genes as effect modifiers of HCMV-associated malignancies is presented below.

## Cytomegalovirus, Gliomas, and GM Alleles

Human cytomegalovirus is not considered an oncogenic virus at present, but several features of HCMV biology overlap with the essential alterations of cell physiology, including tumor microenvironment, that are hallmarks of cancer, as enunciated by Hanahan and Weinberg (9). In 2002, Cobbs and colleagues, for the first time, presented evidence that implicated this virus in gliomagenesis (10). Since then numerous studies have documented the presence of HCMV DNA, RNA, and protein in the majority of human gliomas, and HCMV-based therapies appear promising (11–13).

Although the current consensus in the field is that HCMV is an active promoter or oncomodulator of gliomagenesis, it is clear that among exposed individuals, not everyone is equally likely to develop HCMV-associated glioma, implying the presence of host genetic factors that might modulate immunity to this virus. Ig GM genes are good candidates, as they are involved in immunity to several viruses, including HCMV (14–23). These observations – and especially the modulating influence of GM alleles on HCMV’s immunoevasion strategies discussed above – led us to hypothesize that these polymorphic determinants might be risk factors for the development of glioma (24).

Using a case–control design, we tested this hypothesis in a group of 120 glioma patients and 133 random blood donors as controls. Both patients and controls were unrelated Caucasians from Portugal. DNA from the study subjects was typed for GM 3 and 17 alleles. Genotype frequencies were in Hardy–Weinberg equilibrium in both groups. Odds ratios (OR) for the GM genotypes for the risk of glioma were estimated by unconditional multivariate logistic regression analysis, adjusted for potential confounding variables (patient age and sex). Compared to subjects who were homozygous for the GM 17 allele, the GM 3 homozygotes were over twice as likely (OR = 2.82), and the GM 3/17 heterozygotes were over three times as likely (OR = 3.13) to develop glioma. Likewise, when comparing the combined GM 3/3 + 3/17 (i.e., GM 3-carriers) genotypes to the GM 17/17 genotypes, the presence of the GM 3 allele conferred a significantly increased risk for glioma (OR = 2.95) and high-grade astrocytoma (OR = 3.11).

A multivariate Cox proportional hazard model, adjusted for patient age and sex, was used to investigate the association between GM genotypes and overall survival. Glioblastoma patients carrying the GM 17/17 genotype had longer median overall survival (35 months) than patients carrying the GM 3/17 or GM 3/3 genotypes (16 months), but the difference was not statistically significant, probably a reflection of small sample size (HR = 2.78, *p* = 0.19).

These results support the hypothesis that GM 3 and GM 17 alleles are risk factors for glioma (25). They also shed light on the possible reasons behind the racial differences in the prevalence of glioma. Age-adjusted glioma rates are considerably higher in Caucasians than in people of African descent. As noted earlier, the GM 3 allele is absent or extremely rare in people with unmixed African ancestry.

## Cytomegalovirus and Uncommon GM Genotypes Associated with Neuroblastoma

Almost four decades ago, two very unusual GM genotypes, 1+3+21−5+ and 1+3+21+5−, were shown to be highly significantly (*p* < 0.00005) associated with susceptibility to neuroblastoma, the most common extracranial solid tumor of childhood (26). The mechanism underlying this association has not yet been elucidated. However, a recent report documenting the expression of early and late HCMV proteins in primary neuroblastomas, neuroblastoma cell lines, and neuroblastoma xenografts (27) shed some light on the paradox: how a common virus (prevalence ~80%) could be associated with an uncommon (prevalence ~0.025%) disease. It is noteworthy that both uncommon genotypes associated with neuroblastoma include GM 3, the allele associated with high affinity to the HCMV *TRL11/IRL11*-encoded FcγR. To gain further mechanistic insights into the GM gene involvement in the pathogenesis of this malignancy, it would be necessary to evaluate the affinity of viral FcγRs to the IgG proteins expressing other GM alleles – 1, 5, and 21 – that constitute the genotypes associated with neuroblastoma.

## GM Alleles, Cytomegalovirus, and the Risk of Breast Cancer

Using a matched case–control design, we have evaluated the contribution of GM alleles to the risk of breast cancer in a large (1710 subjects) multiethnic study population from Japan and Brazil (28). The study subjects were typed for several GM alleles – IgG1 (3, 17), IgG2 (23 + ∕−), and IgG3 (5, 21). Conditional logistic regression models were constructed to detect the association between GM alleles and breast cancer.

After adjusting for the potential confounders, the GM 3 allele of IgG1 was significantly associated with susceptibility to breast cancer in Caucasian subjects from Brazil (*p* = 0.0147); subjects possessing this allele were over twice as likely to develop breast cancer as those who lacked this allele (OR = 2.07). No significant associations were found in other population groups. The reasons for racial differences in disease associations are not clear. Linkage disequilibrium between GM alleles in the Japanese is different from that in people of African or European descent, resulting in distinct arrays of GM haplotypes in various groups. It follows that linkage disequilibrium between any putative risk-conferring genes for breast cancer and GM alleles might also be different in these groups, contributing to the ethnic differences in genetic associations. Multiple genetic and non-genetic factors probably contribute to the risk of breast cancer, and racial differences in these factors may contribute to the differences in the observed associations. It is relevant to note that, of the four population groups examined, the frequency of the high affinity (to HCMV–FcγR) GM 3 allele was the highest in the subjects of Caucasian descent (Brazil).

As in glioma and neuroblastoma, increasing evidence implicates HCMV in the etiopathogenesis of breast cancer. Evidence of viral expression has been found in over 97% of neoplastic breast epithelium (29). These findings were confirmed and extended in a recent investigation, which found 100% of the primary breast cancer samples HCMV positive, and also detected viral protein expression in neoplastic cells in sentinel lymph node metastases of breast cancer (30). Our finding of a significant association between the GM 3 allele and susceptibility to breast cancer, which is consistent with the predictions from the IgG1–HCMV–FcγR binding studies (7), appears to unite the putative genetic and viral etiology of this malignancy.

## Other Mechanisms of GM Gene Involvement in Cancer

So far, we have discussed how GM alleles could contribute to the risk of glioma, neuroblastoma, and breast cancer by modulating the viral immunoevasion strategies. However, this is unlikely to be the only mechanism underlying their involvement in these malignancies. These determinants could also contribute to the disease risk by influencing antibody responsiveness to the relevant viral and tumor-associated antigens. In a recent study, we found the levels of IgG antibodies to HCMV glycoprotein B to be the highest in GM 17/17 homozygotes, intermediate in GM 3/17 heterozygotes, and the lowest in GM 3/3 homozygotes (31). If anti-HCMV antibodies were protective, subjects with the low responder genotype – GM 3/3 – would be expected to be at a higher risk of developing HCMV-associated diseases than the subjects with the high responder GM 17/17 genotype, a prediction supported by our finding of GM 3 association with glioma and breast cancer discussed above. We have also reported contribution of GM alleles to humoral immunity to some tumor-associated antigens relevant to these malignancies, e.g., human epidermal growth factor receptor 2 (HER2) and mucin 1 (32, 33).

Although GM allotypes are expressed in the constant region of γ chains, they could influence antibody specificity and affinity by imposing structural constraints (conformation) on the variable region. It is relevant to note that amino acid sequence polymorphism in the CH1 domain of the γ1 chain – where the allelic determinants GM 3 and GM 17 are located – has been shown to modulate the kinetic competence of antigen binding sites (34). Another mechanism of GM gene involvement in cancer pathogenesis could involve ADCC, a major host immunosurveillance mechanism against tumors and virally infected cells. IgG antibody mediated ADCC is triggered upon ligation of FcγR to the Fc region of IgG molecules, where the majority of GM alleles are expressed. It follows that genetic variation in FcγR and Fc could contribute to the differences in the magnitude of ADCC. We have presented evidence of interactive effects of GM and FcγRIIIa alleles on ADCC of cancer cells overexpressing HER1, HER2, mucin 1, and the disialoganglioside antigen GD2 (35–37).

## GM Genes and Genome-Wide Association Studies

Genome-wide association studies (GWAS) do not evaluate GM genes and, therefore, none of the GM gene–disease associations that were identified by hypothesis driven candidate gene approaches have been confirmed or refuted by these studies. GM genes are not included in the genotyping platforms commonly used in GWAS. Since these genes were not typed in the HapMap and 1000 Genomes projects, they cannot be imputed. Genes encoding IgG subclasses that harbor GM alleles are highly homologous and apparently not amenable to the high throughput genotyping technologies. This attribute may have contributed to their exclusion from the genotyping panels.

## Future Studies

For a thorough understanding of the underlying immunogenetic mechanisms in HCMV-associated diseases, further studies are warranted. A brief outline of some of these follows. The IgG–HCMV–FcγR binding studies discussed above employed the HCMV strain AD169 for cloning the *TRL11* sequences. Whether or not *TRL11*-encoded FcγR from another strain would have the same affinity for allotypically disparate IgG1 proteins needs to be investigated. Additional studies to measure binding between allotypically different IgG2 and IgG3 proteins and HCMV *TRL11-* and *UL119*-encoded FcγRs are warranted. In this context, it would be interesting to evaluate the recently described HCMV *RL13*-encoded FcγR (38) for its binding affinity to allotypically disparate IgG proteins.

In view of the strong linkage disequilibrium between particular GM alleles within a racial group, all GM alleles that constitute racially associated haplotypes must be evaluated to obtain a more complete understanding of the contribution of GM alleles to the risk of malignant diseases discussed above. Future studies should also consider examining possible interactive contribution of particular candidate genes to the risk of developing glioma, neuroblastoma, and breast cancer. Genes do not act in isolation: there is increasing evidence that epistasis – modification of the action of a gene by one or more other genes – plays a significant role in human diseases (39–41). GM and HLA are excellent candidate genes for investigations of epistasis in these malignancies, as both are targets of HCMV immunoevasion strategies (7, 42) and have been associated with the risk of these cancers (25, 26, 28, 43–45). Results of these investigations may identify novel pathways to cancer immunity, which would be helpful in devising effective immunotherapeutic strategies against these malignancies.

## Conflict of Interest Statement

The author declares that the research was conducted in the absence of any commercial or financial relationships that could be construed as a potential conflict of interest.
